# Foliar application of yeast extract mitigates water deficit stress and elicits hypericin and phenolic production in *Hypericum perforatum* L

**DOI:** 10.1038/s41598-025-06013-w

**Published:** 2025-07-01

**Authors:** Rayhaneh Amooaghaie, Nafiseh Rajaie

**Affiliations:** 1 Plant Science Department, Faculty of Science, Shahrekord University, Shahrekord, Iran; 2 Biotechnology Research Institute, Shahrekord University, Shahrekord, Iran

**Keywords:** Antioxidant enzymes, Drought stress, FRAP, Phenolics, DPPH, St john’s wort, Physiology, Plant sciences

## Abstract

Yeast extract has emerged as a bio-elicitor capable of modulating secondary metabolism and stress tolerance in plants, but its impact on St John’s Wort (*Hypericum perforatum* L.) remains unexplored. Therefore, the interactive effects of yeast extract (0, 3, and 6 g L^−1^) and irrigation intervals (7, 10, and 13 days) on hypericin and phenolic production in this medicinal herb were investigated in a field experiment. The prolonged irrigation intervals decreased biomass during both seasons. Hypericin content peaked under the 10-day irrigation interval but declined in the 13-day irrigation interval. Foliar spraying of yeast extract improved biomass, chlorophyll *a*,* b*, carotenoids, relative water content, and hypericin concentration across all water regimes. Yeast extract application reduced hydrogen peroxide and malondialdehyde contents in water deficit-subjected plants due to increased activity of superoxide dismutase and catalase, and elevated levels of total phenol and flavonoid contents in the leaves. The highest contents of hypericin and phenolics were recorded with applying 6 g L^−1^ yeast extract under the 10-day irrigation interval, corresponding with the strongest 2,2-diphenylpicrylhydrazyl scavenging activity and ferric-reducing power in the leaves. These findings suggest that yeast extract spraying might be a promising approach for enhancing the productivity and quality of medicinal plants under water deficit.

## Introduction

Over the past few decades, a growing global demand for natural remedies has prompted significant interest in enhancing the productivity and bioactive compounds of medicinal plants^1^. St. John’s Wort (*Hypericum perforatum* L.) is a medicinal herb of the Hypericaceae family that has garnered significant attention due to its diverse bioactive compounds and extensive range of pharmacological activities^2,3^. Research on this perennial herb has primarily focused on naphthodianthrones like hypericin and pseudohypericin, as well as phloroglucinols, such as hyperforin, which are largely credited for their antidepressant properties^4^. However, St. John’s Wort is also rich in phenolic compounds, including chlorogenic acids, caffeic acid, and xanthones, as well as various flavonoids such as rutin, quercetin, quercitrin, hyperoside, isoquercitrin, and procyanidins^5^. Recent studies have highlighted the potential of St. John’s Wort in exhibiting antimalarial, antifungal, anti-inflammatory, diuretic, analgesic, wound-healing, and sedative effects^2^. Owing to these therapeutic properties—especially its antidepressant effects—there is an increasing industrial interest in developing standardized production methods and enhancing the herb’s bioactive compound content, particularly hypericin and phenolic compounds^5^.

Secondary metabolites especially phenolic compounds are crucial for plant adaptation to varying environmental conditions; their production is often triggered by various biotic and abiotic stresses^6–10^. Among abiotic stresses, water scarcity stands out as one of the most pervasive global environmental challenges, negatively impacting the morphology, physiology, and productivity of crops and medicinal plants^11^. Water deficit not only reduces osmotic potential but also increases the production of reactive oxygen species (ROS) in plant cells. These ROS cause oxidative damage to cellular membranes and disrupt critical biochemical processes such as photosynthesis and ATP synthesis^12,13^. To counteract these effects, plants activate antioxidative enzymes and biosynthesis of antioxidant secondary metabolites to enhance ROS scavenging^11,13^. While mild stressful conditions may boost secondary metabolism in medicinal plants, severe stresses can negatively impact photosynthesis and reduce both the quantity and quality of secondary metabolites^7,14–16^. Therefore, developing practical solutions to maximize plant growth and secondary metabolite production under stress conditions is essential.

Among various approaches, the application of natural biostimulants has emerged as a promising technology to enhance stress tolerance and secondary metabolism in plants^1,17^. One of the natural elicitors is yeast extract which the cost-effectiveness and safety make it an attractive option for agricultural systems^18–20^. Yeast extract is a rich source of amino acids, carbohydrates, oligosaccharides, minerals, vitamins, and phytohormones^21–23^. These components stimulate plant growth and secondary metabolism without posing health risks to humans^24,25^. Yeast extract has also been shown to improve plant stress tolerance through various physiological and biochemical mechanisms. Several studies have shown that foliar application of yeast enhances stress tolerance in water deficit –exposed cowpea^26^ drought-stressed wheat^27^ and salt-subjected lettuce^28^ plants. El-Tohamy et al.^29^ noted that yeast treatments (1.5 g L^−1^), either alone or combined with GA_3_, enhanced relative water content and total chlorophyll in sweet potato leaves while boosting productivity and soluble solid content in roots grown in sandy soils. Yeast extract has been primarily applied as an elicitor in plant cell, tissue, and organ cultures to stimulate the production of pharmaceutically valuable compounds in medicinal plants. For instance, yeast extract supplementation has been shown to boost the production of azadirachtin in *Azadirachta indica*^30^ rosmarinic acid in *Agastache rugosa* in cell suspension cultures^31^ and increased the biosynthesis of mangiferin, amarogentin flavonoids and total phenolic contents in *Swertia chirata* in vitro callus cultures^32^. In addition, yeast extract enhanced rutin and quercetin derivatives, tannins, anthocyanins, total flavonoids, total phenol content, and antioxidant activity in *Oryza sativa* L. cell suspensions^33^. Yeast extract supplementation significantly enhanced phenylalanine ammonia-lyase (PAL) and tyrosine ammonia-lyase (TAL) activities, and increased flavonoids and phenolic acids in *Zataria multiflora* cell suspension^34^. Zaman et al.^35^ reported that yeast extract increased total phenolic and flavonoid contents as well as enhanced antioxidant activities such as ABTS (2,2 azinobis 3-ethylbenzthiazoline-6-sulphonic acid), FRAP (ferric-reducing antioxidant power) and DPPH (2,2-diphenyl-1-picryhydrazyl) in callus culture of purple basil (*Ocimum basilicum* L. var *purpurascens*).

Despite the above-mentioned promising findings, there is limited research on the effects of foliar application of yeast extract on secondary metabolism in medicinal plants. Youssef et al.^24^ reported that yeast treatments (1.5 g L^−1^), with or without GA_3_, enhanced growth parameters, photosynthetic pigments, NPK contents, and the levels of total phenolics, flavonoids, soluble sugars, and glycosides in *Solidago virgaurea* grown in alkaline soils. El-Serafy et al.^36^ found that foliar yeast sprays promoted growth parameters, increased photosynthetic pigment levels, N, Mg, P, Ca, K, and total phenol content in fennel, along with altering key essential oil components. Saad-Allah et al.^37^ also revealed that yeast application improved total chlorophyll content, PSII photochemical efficiency, and silymarin production in *Sylibum marianum* L. While these studies are encouraging, further research is needed to fully elucidate the potential of yeast extract for improving the quality and quantity of medicinal plants under various stresses. In addition, investigating the long-term impacts of yeast extract on secondary metabolism and determining its optimal concentration for specific medicinal plants such as St John’s Wort is crucial for maximizing benefits.

The antidepressant and antioxidant properties of St John’s Wort are primarily due to the presence of bioactive compounds like hypericin and various phenolic compounds found in its flowering tops^2^. Hypericin is a naphthodianthrone that synthesized essentially by some *Hypericum* spp., and its concentration in the flowering tops is considered as a quality marker for *H. perforatum*. Furthermore, hypericin exhibits antiviral, antibacterial, and antioxidant activities and as a promising photosensitizer is applied in photodynamic diagnosis and cancer therapy^5^. The exploration of hypericin’s potential applications, alongside the benefits of phenolic compounds for human health, has increased the application of St John’s Wort flowering tops in both traditional medicine and the pharmaceutical industry. However, the extensive harvesting of wild sources of *H. perforatum* has significantly reduced its natural population, posing challenges in meeting market demands and highlighting the need to expand its cultivation^2^. St. John’s Wort has a robust ability to endure harsh conditions, including drought stress^3^, making it a viable candidate for cultivation in arid and semi-arid regions like Iran. However, cultivation of this herb often faces instability in yield and fluctuations in active ingredient concentration, especially under abiotic stresses^5^. For instance, it has been shown that prolonged water deficits can negatively impact both the productivity and phytochemical composition of the plant^16,^^38^. Therefore, finding innovative solutions for maximizing hypericin and phenolic production in this herb and enhancing its resilience to various stresses is a critical objective for ongoing research. The documented benefits of yeast in enhancing stress tolerance and secondary metabolism in edible crops^19,26,39^ this should be 40enthused us to conduct a long-term field experiment to investigate the effects of foliar application of yeast extract on St John’s Wort. To the best of our knowledge, the impact of yeast on secondary metabolism and stress tolerance of *H. perforatum* has not yet been explored. The current study for the first time explored the effects of foliar application of yeast extract on antioxidant responses as well as hypericin and phenolic contents as quality markers of this herb under varying water regimes.

## Materials and methods

### Plant cultivation and performance of the experiment

St. John’s Wort (*Hypericum perforatum* L.) seeds were obtained from Pakan Bazr Company in Isfahan, Iran. Initially, seeds were sown in pots filled with cocopeat and perlite in a greenhouse, and seedlings were transplanted to an open field within the research farm of Gol Daru Company at Kelishad, Iran (x = 550043, Y = 3598801). Soil characteristics included pH 6.95, 0.2% organic carbon, 0.02% total nitrogen, 16.5 mg kg^−1^ available phosphorus, 205 mg kg^−1^ available potassium, and electrical conductivity of 1.5 dS m^−1^.In the first year (2013), all plots were watered every 7 days throughout spring and summer, from transplanting to full flowering time (harvest time), to fully meet the water requirements of the plants. Then, aerial parts of the plants were cut, while roots remained for regrowth the following year. No treatment or test was performed on these plants. It is noteworthy that the biomass yield of flowering tops in the first year of cultivation is relatively low. Based on the natural growth pattern, St. John’s Wort plants achieve their mature size and morphology by the second year, which aligns with typical increases in flowering tops and hypericin content in this herb^40^. Therefore, the current study was conducted in the second year.

In the second year of plant growth (i.e., 2014), the regrowth process was occurred in two distinct seasons. The first season commenced in late February and concluded on June 15th. The second season spanned from June 17th to September 30th. Therefore, two harvests were executed, on June 15th and September 30th. At the start of both seasons, 200 kg ha^−1^ of Urea and 20 kg ha^−1^ of NPK fertilizer (20-20-20) were applied to support the regrowth of plants. The climatic characteristics recorded at the location of the experiment during the two growing seasons are presented in Table 1.

**Table 1:** Monthly average temperature, precipitation, and relative humidity during February-September 2014.

	February	March	April	May	June	July	August	September
Temperature	5.5	10.1	14	19.2	24.4	29.2	28.5	24.1
Precipitation	10	57.2	16.3	11.8	0.7	0.0	0.0	0.0
Humidity	59	46	39	41	28	21	24	25

A split-plot experiment was conducted with a randomized complete block design (RCBD) and three replications. Various irrigation intervals were implemented in the main plots, and foliar spraying with different concentrations (0, 3, and 6 g L^−1^) of yeast extract was performed in the subplots according to statistical design. Based on the report of the Agricultural Research Center of Iran, normal irrigation for this plant is every 7 days. Therefore, various irrigation intervals in the main plots were included: every 7, 10, and 13 days, which respectively corresponded with 83–86% field capacity, 69–73% F.C., and 48–52% F.C., as evidenced by measuring soil moisture with a TDR device. These intervals were considered as normal irrigation, moderate water deficit, and severe water deficit in this study. It is worth noting that yeast extract concentrations were chosen based on previous studies^26,36^ and active yeast extract (*Saccharomyces cerevisiae*) was prepared by dissolving a required quantity of dry yeast in distilled water; a 1:1 ratio of sugar was added (as a source of C and N). The yeast culture was kept overnight before application to the plants for activation and the formation of beneficial bioactive components in them^41^.

In the first season of the second year of plant growth (i.e., 2014), plants resumed vegetation in late February. Water deficit was not implemented in the first month of the growth cycle, and all plots were irrigated at 7-day intervals. On 30 March, when plants reached an average height of 30 cm, irrigation regimes and foliar spraying with yeast extract were initiated simultaneously. The foliar spraying with yeast extract was carried out three times: the first on 30 March during the early vegetative stage, the second on 15 May before the flowering stage, and the third on 1 June when plants were in the 25% flowering stage. The yeast extract solutions (100 mL/plant) were applied using a hand sprayer on aerial parts of the plants. One day after the final yeast extract spray, fresh leaves were collected, immediately frozen in liquid nitrogen, and stored at − 80 °C for various biochemical analyses. Then, all plants were cut at ground level at the full flowering stage on 15 June. Samples of five plants were randomly taken per treatment from the middle of plots and were air-dried in the shade for one week at room temperature. To determine the herbal yields, the average dry weight of the aerial parts of 5 plants was reported as biomass per plant.

In the second season of plant growth in 2014, plants commenced vegetation after the first cutting. To facilitate quick regrowth, all plots were irrigated twice at a 7-day interval. Then, plants grew again under the same irrigation regimes, and foliar spraying with yeast extract was performed three times at 30, 55, and 70 days after regrowth. Finally, plants were harvested on 30 September 2014 for the second time, and after drying, biomass and hypericin content were measured.

### Evaluation of water status in leaves

To determine the relative water content (RWC) in fresh leaves the following equation was applied:

$$\:\text{\%}\text{R}\text{W}\text{C}=\left[\frac{(\text{F}\text{W}-\text{D}\text{W})}{(\text{T}\text{W}-\text{D}\text{W})}\right]\times\:100$$

Where DW is the dry weight (after drying in an oven at 75 °C), FW is fresh weight, and turgor weight is the weight of leaf samples after floating the leaves in water for 7 h^15^.

### Measurement of chlorophyll and carotenoids

The fresh leaf samples were macerated using acetone (80%, v/v), and after filtering and diluting the absorbance of the extract was read at 663, 645, and 470 nm. Then, the content of chlorophyll *a*,* b* (Chl *a* and Chl *b*), and carotenoids were calculated using following formula suggested by Lichtenthaler and Wellburn^42^.

$$\:\text{C}\text{h}\text{l}\text{o}\text{r}\text{o}\text{p}\text{h}\text{y}\text{l}\text{l}\:a=\frac{\left[\left(12.7\times\:D663\right)-\left(2.69\times\:D645\right)\right]\times\:V}{1000\times\:W}$$

$$\:\text{C}\text{h}\text{l}\text{o}\text{r}\text{o}\text{p}\text{h}\text{y}\text{l}\text{l}\:b=\frac{\left[\left(22.9\times\:D645\right)-\left(4.93\times\:D663\right)\right]\times\:V}{1000\times\:W}$$

$$\:\text{C}\text{a}\text{r}\text{o}\text{t}\text{e}\text{n}\text{o}\text{i}\text{d}\text{s}=\frac{\left(100\times\:D470-1.82\times\:\text{C}\text{h}\text{l}.\:\text{a}-85.02\times\:\text{C}\text{h}\text{l}.\text{b}\right)}{198}$$

### Estimation of hydrogen peroxide and malondialdehyde contents

Heath and Packer’s method was adopted to measure malondialdehyde (MDA) content as an indicator of membrane damage. Leaf samples were macerated in trichloroacetic acid and centrifuged at 10,000 ×g for 5 min. The mixture of thiobarbituric acid, tricholoroacetic acid, and supernatant, was incubated and centrifuged. The MDA content was computed by reading the optical density at 600 and 532 nm and using an extinction coefficient of 155 mM^−1^ cm^−1^.

H_2_O_2_ content was quantified using a KI reagent using the procedure adopted by Velikova et al.^44^. The H_2_O_2_ content was calculated by recording the optical absorbance at 390 nm and using a standard curve.

### Assaying the antioxidant enzyme activities

First, frozen leaves were homogenized using an extraction buffer and centrifuged. Then, superoxidase dismutase (SOD) and Ascorbate peroxidase (APX) activity in the resulting supernatant was determined by the method adopted by Nabaei et al.^10^. SOD activity was assayed by recording a 50% decrease in absorbance at 560 nm, indicating a 50% inhibition in the reduction of nitro blue tetrazolium (NBT) induced by the enzyme. Ascorbate peroxidase (APX) activity was assayed based on reading absorbance values of the reaction mixture at 290 nm at 5-sec intervals for 1 min and was calculated using the extinction coefficient of 2.8 mM^−1^ cm^−1^. Catalase (CAT) activity was evaluated based on the decrease in optical absorbance at 240 nm per minute due to the decomposition of H_2_O_2_ and using the extinction coefficient of 39.4 Mm^−1^cm^−1^^7^.

### Quantification of hypericin content

First, plant samples were dried in the shade and then, powdered and soaked in acidic acetone for 2 h. The resulting solution was filtrated twice and then dried by a vacuum dryer. The residue was dissolved and diluted to 100 ml with methanol. The absorbance of the solution is read at 587 nm compared to methanol as blank. Hypericin content was calculated by the following equation^45^.

$$Hipg\% =\frac{A}{{780}} \cdot \frac{{100}}{m}$$

where A is the optical absorbance of the extract, m is the weight (g) of the sample applied for extraction, and 780 is the specific absorbance of hypericin at 587 nm.

### Quantification of total phenolic content and total flavonoid content

To measure phenol content, 1 g of powdered and dried leaves was soaked in methanol 80% and 10 min centrifuged (10000 ×g) at 4 °C. After 15 min incubating the mixture of deionized water, supernatant, Folin-Ciocaltu reagent and sodium carbonate 2%, the absorbance of the solution was read at 750 nm. Total phenol content (TPC) was expressed as mg gallic acid equivalent per gram dry weight^9^.

To quantify total flavonoid content (TFC), the supernatant was mixed with glacial acetic acid, and AlCl_3_ for 30 min, and the optical absorbance of a flavonoid–aluminum complex was read at 415 nm. TFC is expressed as mg catechin equivalents per gram dry weight^15^.

### **DPPH scavenging activity and ferric-reducing power (FRAP) assay**

The scavenging capacity of DPPH radicals was assayed using the method adopted by Amooaghaie et al.^15^. In the first, leaf samples were extracted in methanol, and 10 min centrifuged (10000 ×g) and the supernatant was used for measuring DPPH radical scavenging capacity. After 1 h of incubating the mixture of extract and DPPH solution (0.004%) in methanol in a dark room, the discoloration was estimated at 517 nm. The DPPH scavenging capacity of the extracts was computed by the following equation:

$${\text{DPPH radical scavenging activity }}\left( \% \right){\text{ }}={\text{ }}[({\text{Abs control}}-{\text{Abs sample}})\left] {/\left( {{\text{Abs control}}} \right)} \right] \times {\text{1}}00$$

To assay the ferric reducing power (FRAP), the extracts were mixed with FRAP reagent containing sodium phosphate buffer, FeCl_3_, and TCA by the method adopted by Benzie and Strain^46^. Then, optical absorbance was read at 700 nm and FRAP values were calculated using a standard curve.

### Statistical analysis

The experiment was conducted as a split-plot experiment with a randomized complete block design (RCBD) and three replications. For ANOVA analysis, SAS software was used, and the comparison of the means was done by Duncan’s multiple range tests at *P* < 0.05. For better comprehension, a correlation heat map based on the Pearson correlation coefficient and hierarchical cluster analysis (HCA) between treatments and variables were performed using R software (ver. 3.5.0, http://www.r-project.org).

## Results

### **Interactive effect of yeast extracts and water regimes on biomass**

**Fig. 1:**
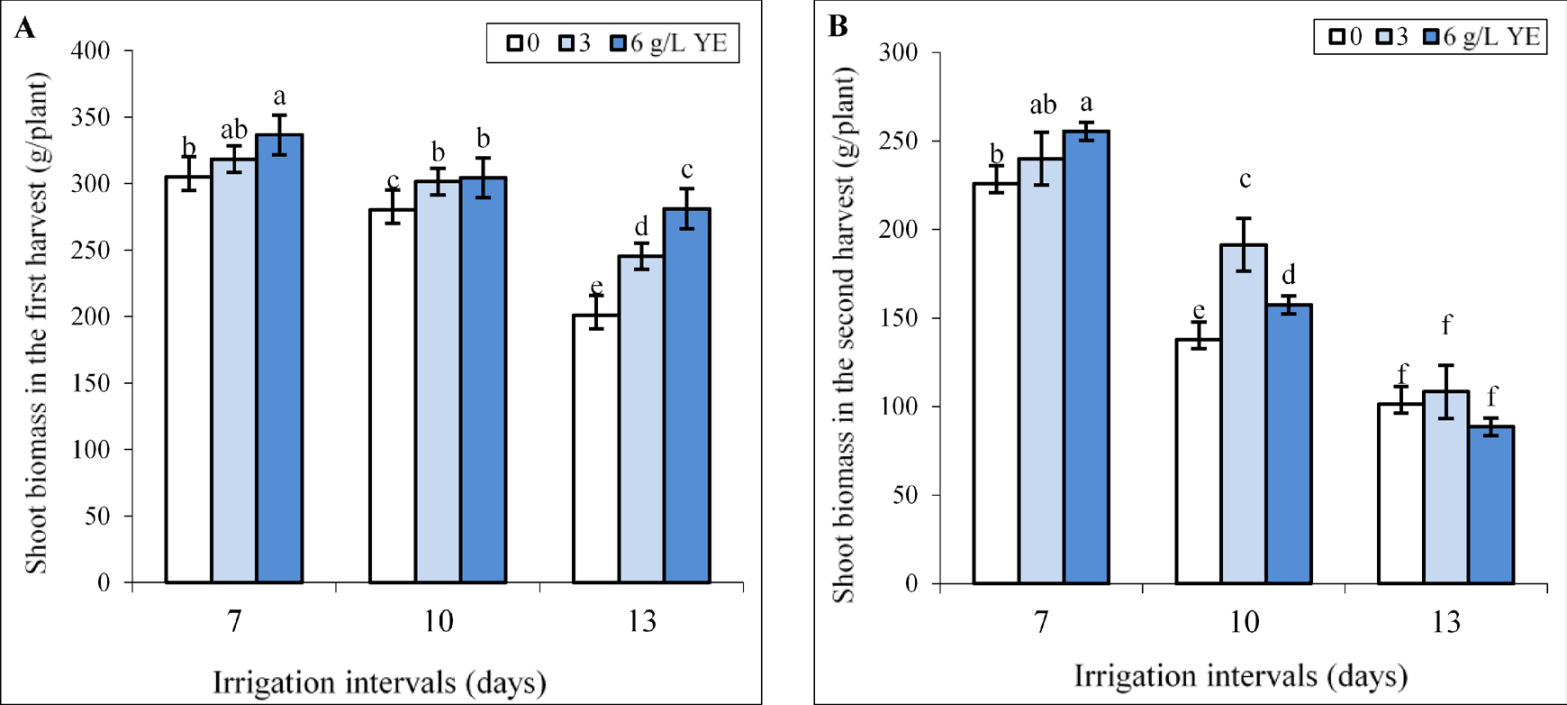
The effect of foliar-applied yeast extract (0, 3, and 6 g L^−1^) on the biomass of *Hypericum perforatum* in two harvests (**A**, **B**) under various irrigation intervals (7, 10, and 13 days). Values with the same letter have no significant difference at *P* ≤ 0.05 based on Duncan’s multiple-range tests.

In the first harvest, spraying with 3 g L^−1^ yeast extract did not change, and the concentration of 6 g L^−1^ significantly increased biomass (10.5%) under normal (7-day interval) irrigation (Fig. 1A). The results showed that increasing irrigation intervals from 7 to 10 (mild water deficit) and 13 days (severe water deficit) reduced biomass by 8.13% and 34.45% respectively in the first harvest. Foliar application of 3 and 6 g L^−1^ yeast extract notably increased biomass under both reduced irrigation levels. Under mild water deficit, the impact of both yeast extract concentrations on biomass was equal whereas spraying with 6 g L^−1^ more than 3 g L^−1^ yeast extract improved biomass under severe water deficit (Fig. 1A). Foliar spraying with 6 g L^−1^ increased biomass by 10.53% and 39.9% compared to respective controls under mild and severe water deficit.

The negative impact of reduced irrigation was more pronounced in the second harvest than in the first harvest, and mild and severe water deficit decreased biomass by 39% and 55.2% respectively. Foliar spraying with 6 g L^−1^ yeast extract significantly increased biomass under normal (7-day interval) irrigation. However, the concentration of 3 g L^−1^ yeast extract was the best treatment, and more than 6 g L^−1^ increased biomass under the 10-day irrigation interval. Under severe water deficit, the biomass of plants sprayed with both yeast extract concentrations did not significantly change in comparison to the respective control (Fig. 1B).

### **Interactive effect of yeast extracts and water regimes on relative water content**

**Fig. 2:**
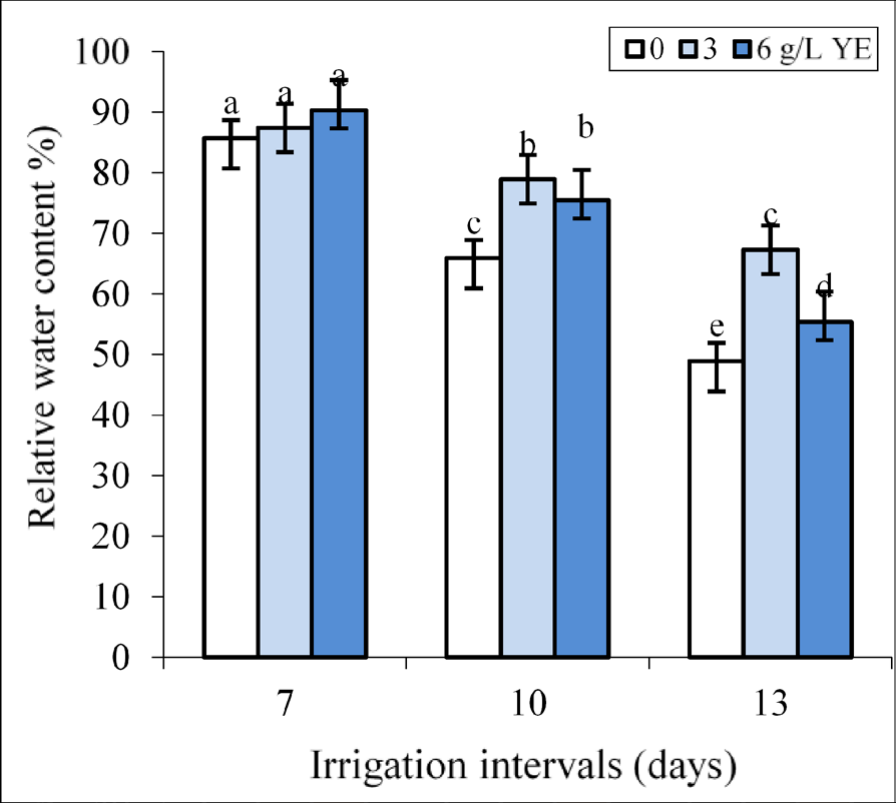
The effect of foliar-applied yeast extract (0, 3, and 6 g L^−1^) on the relative water content of *Hypericum perforatum* under various irrigation intervals (7, 10, and 13 days). Values with the same letter have no significant difference at *P* ≤ 0.05 based on Duncan’s multiple-range tests.

Under normal irrigation (7-day interval), the impact of both concentrations of yeast extract on RWC was insignificant (Fig. 2).

By increasing the irrigation intervals to 10 and 13 days, the concentrations of 3 and 6 g L^−1^ of yeast extract significantly increased RWC compared to respective controls in each group. Under mild water deficit, the impact of both yeast extract concentrations on RWC was equal whereas spraying with 3 g L^−1^ more than 6 g L^−1^ yeast improved RWC under severe water deficit. Foliar spraying with 6 g L^−1^ increased RWC by 14.56% and 15.29% compared to respective controls under mild and severe water deficit.

### **Interactive effect of yeast extracts and water regimes on photosynthetic pigments**

**Fig. 3:**
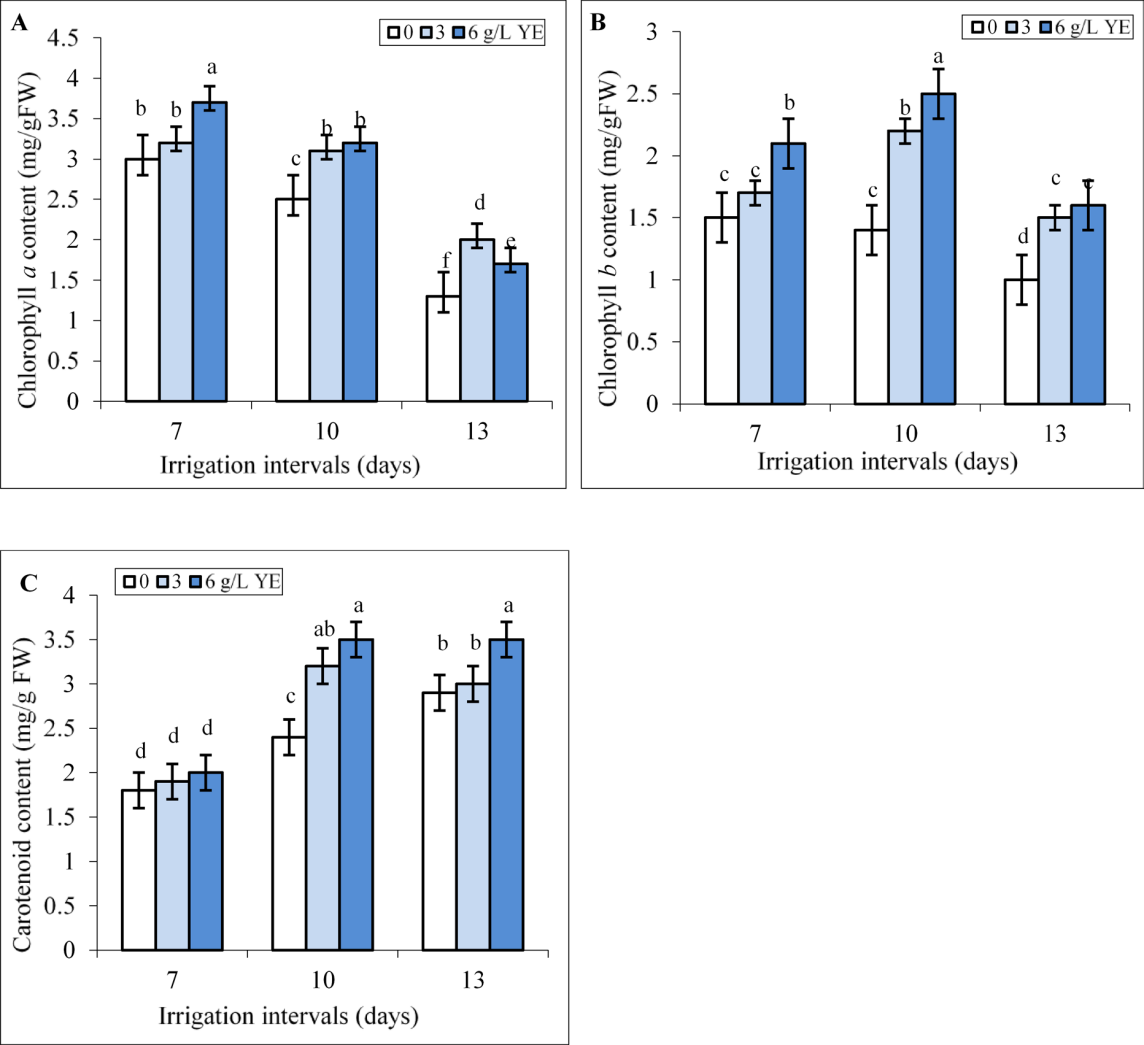
The impact of foliar-applied yeast extract (0, 3, and 6 g L^−1^) on chlorophyll *a* (**A**), chlorophyll *b* (**B**), and carotenoid (**C**) contents in leaves of *Hypericum perforatum* under various irrigation intervals (7, 10, and 13 days). Values with the same letter have no significant difference at *P* ≤ 0.05 based on Duncan’s multiple-range tests.

Under normal irrigation (7-day-interval), the concentration of 6 g L^−1^ yeast extract significantly increased Chl *a*, and Chl *b* contents whereas the effect of spraying with 3 and 6 g L^−1^ yeast extract on carotenoid content was insignificant (Fig. 3A-C). The mild and severe water deficit decreased Chl *a* level by 16.6% and 56.7% and Chl *b* content by 6.6% and 33.3% compared to the controls. Foliar spraying with 3 and 6 g L^−1^ yeast extract increased Chl *a*, and Chl *b* contents under mild and severe water deficit, compared to respective controls in these groups (Fig. 3A, B). Foliar spraying with 6 g L^−1^ increased Chl *a* level by 28% and 30.7% and Chl *b* content by 78.5% and 60.3% compared respective controls under mild and severe water deficit.

Our results showed that by increasing the irrigation intervals to 10 and 13 days the content of carotenoids significantly increased compared to the control (Fig. 3C). Under mild water deficit, both concentrations of yeast extract increased the content of carotenoids compared to the respective control. Under severe water deficit, only spraying with the concentration of 6 g L^−1^ yeast extract significantly increased the content of carotenoids compared to the respective control (Fig. 3C). Foliar spraying with 6 g L^−1^ increased the content of carotenoids by 45.8% and 20.6% compared to respective controls under mild and severe water deficit.

### **Interactive effect of yeast extracts and water regimes on MDA and H**_**2**_**O**_**2**_**in the leaves**

**Fig. 4:**
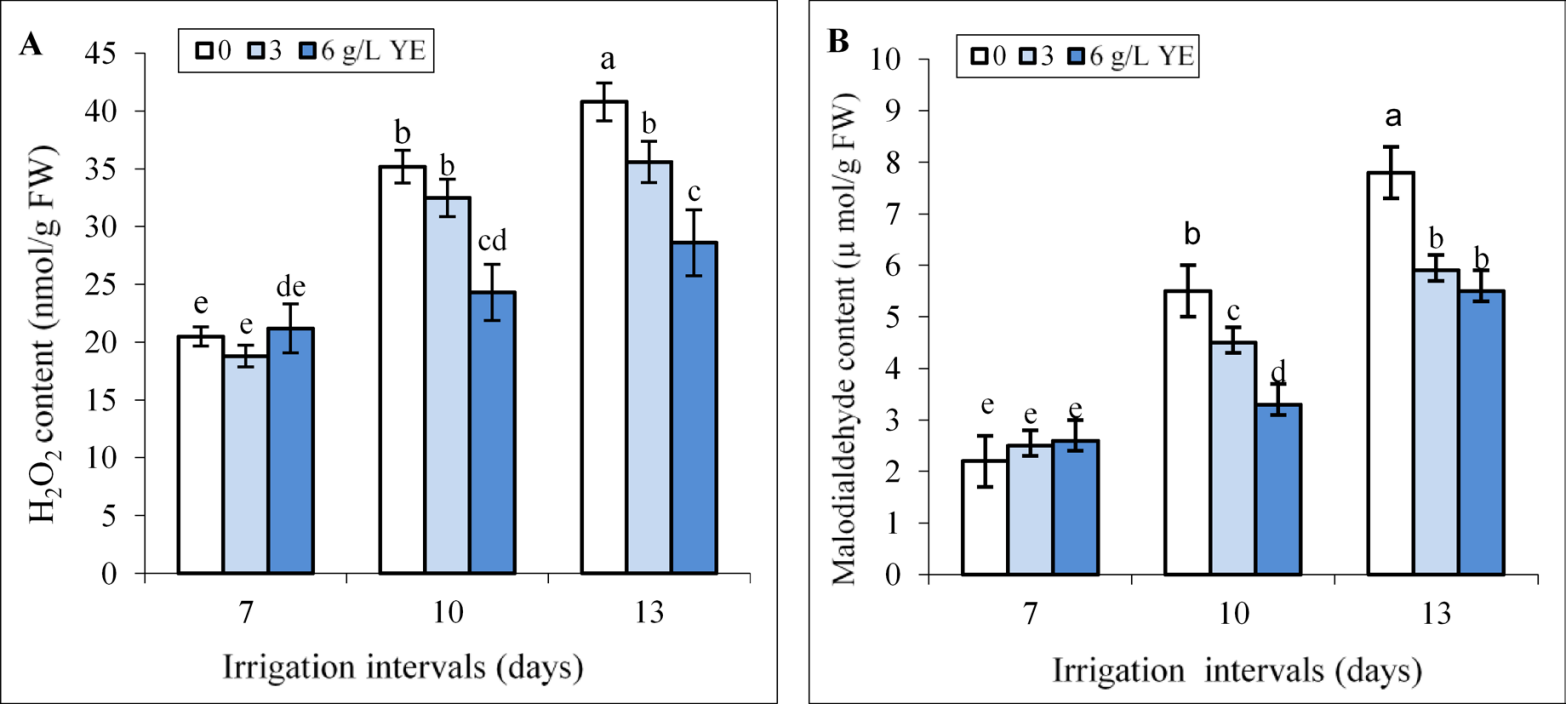
The impact of foliar-applied yeast extract (0, 3, and 6 g/L) on H_2_O_2_ (**A**) and MDA (**B**) contents in leaves of *Hypericum perforatum* under various irrigation intervals (7, 10, and 13 days). Values with the same letter have no significant difference at *P* ≤ 0.05 based on Duncan’s multiple-range tests.

Under normal irrigation (7-day interval), the impact of 3 and 6 g L^−1^ yeast extract on H_2_O_2_ and MDA contents was insignificant compared to control (Fig. 4A, B).

The content of MDA and H_2_O_2_ significantly augmented in leaves under both reduced irrigation levels. Foliar application of 3 and 6 g L^−1^ yeast extract significantly decreased MDA and H_2_O_2_ contents in both reduced irrigation levels, except the effect of 3 g L^−1^ yeast extract on H_2_O_2_ under mild water deficit that was insignificant compared to the respective control. Foliar spraying with 6 g L^−1^ more than 3 g L^−1^ yeast extract decreased H_2_O_2_ and MDA contents in leaves under both water deficit levels (Fig. 4A, B). Foliar spraying with 6 g L^−1^ decreased H_2_O_2_ level by 29% and 30.9% and MDA content by 40% and 29.3% compared to respective controls under mild and severe water deficit.

### Interactive effect of yeast extract and water stress on antioxidant enzyme activity

**Fig. 5:**
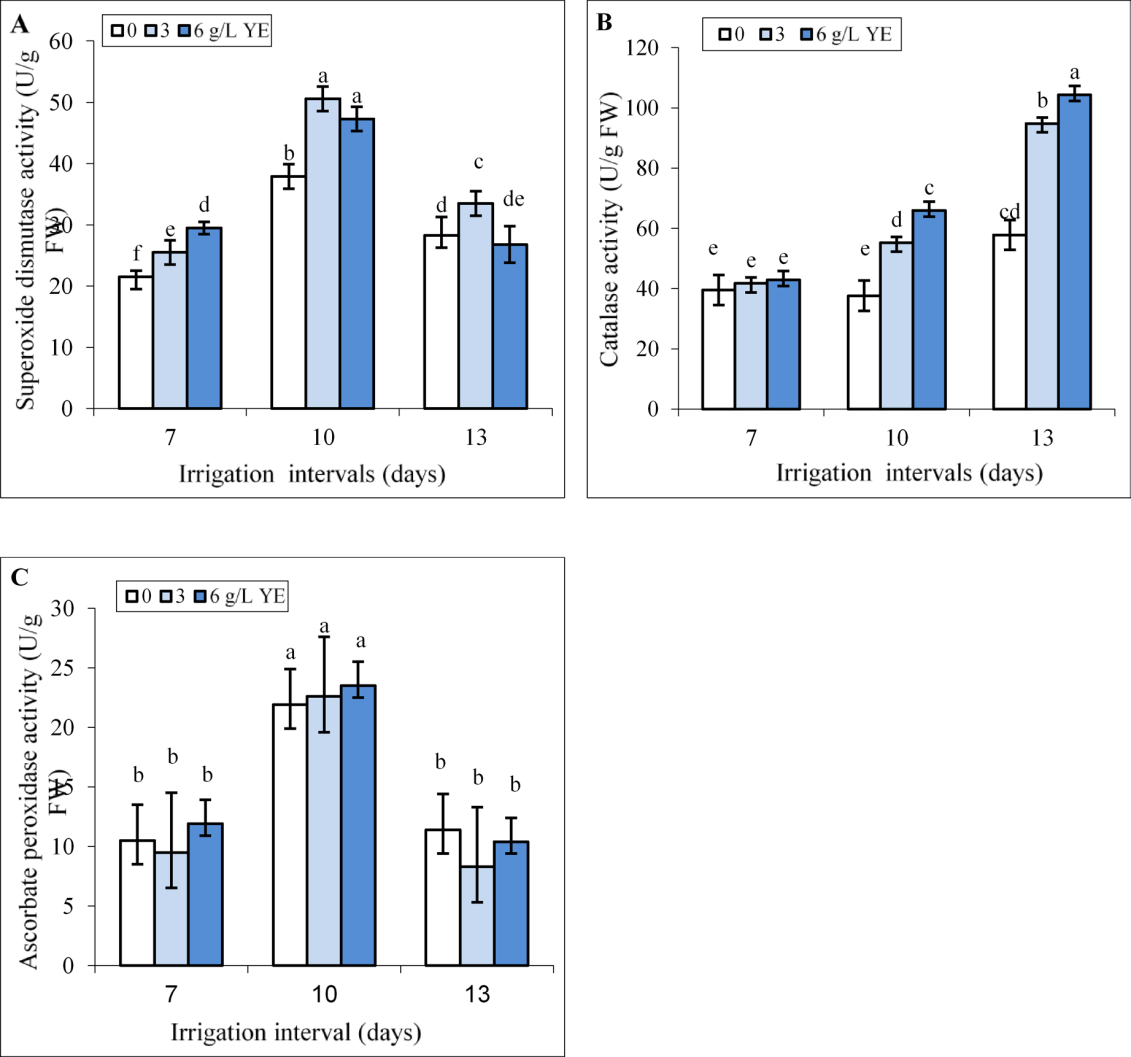
The impact of foliar-applied yeast extract (0, 3, and 6 g L^−1^) on SOD (**A**), CAT (**B**), and APX (**C**) activity in leaves of *Hypericum perforatum* under various irrigation intervals (7, 10, and 13 days). Values with the same letter have no significant difference at *P* ≤ 0.05 based on Duncan’s multiple-range tests.

Under normal irrigation (7-day interval), spraying with 3 and 6 g L^−1^ yeast extract did not change CAT and APX activity, while these treatments slightly increased SOD activity compared to the control (Fig. 5A-C). The CAT activity did not change but SOD and APX activity increased by 76.2% and 2 fold in leaves under mild water deficit. However, the activities of SOD and CAT enzymes were significantly higher than control under severe water deficit. While foliar spraying with 6 g L^−1^ yeast extract increased SOD activity by 27% under mild stress, its effect on this enzyme was insignificant compared to the respective control under severe water deficit (Fig. 5A, B). Foliar spraying with 6 g L^−1^ increased CAT activity by 75.26% and 1.8 fold compared to respective controls under mild and severe water deficit. The impact of spraying with both concentrations of yeast extract on APX activity was insignificant compared to the respective controls under all irrigation intervals (Fig. 5C).

### **Interactive effect of yeast extracts and water regimes on hypericin concentration**

**Fig. 6:**
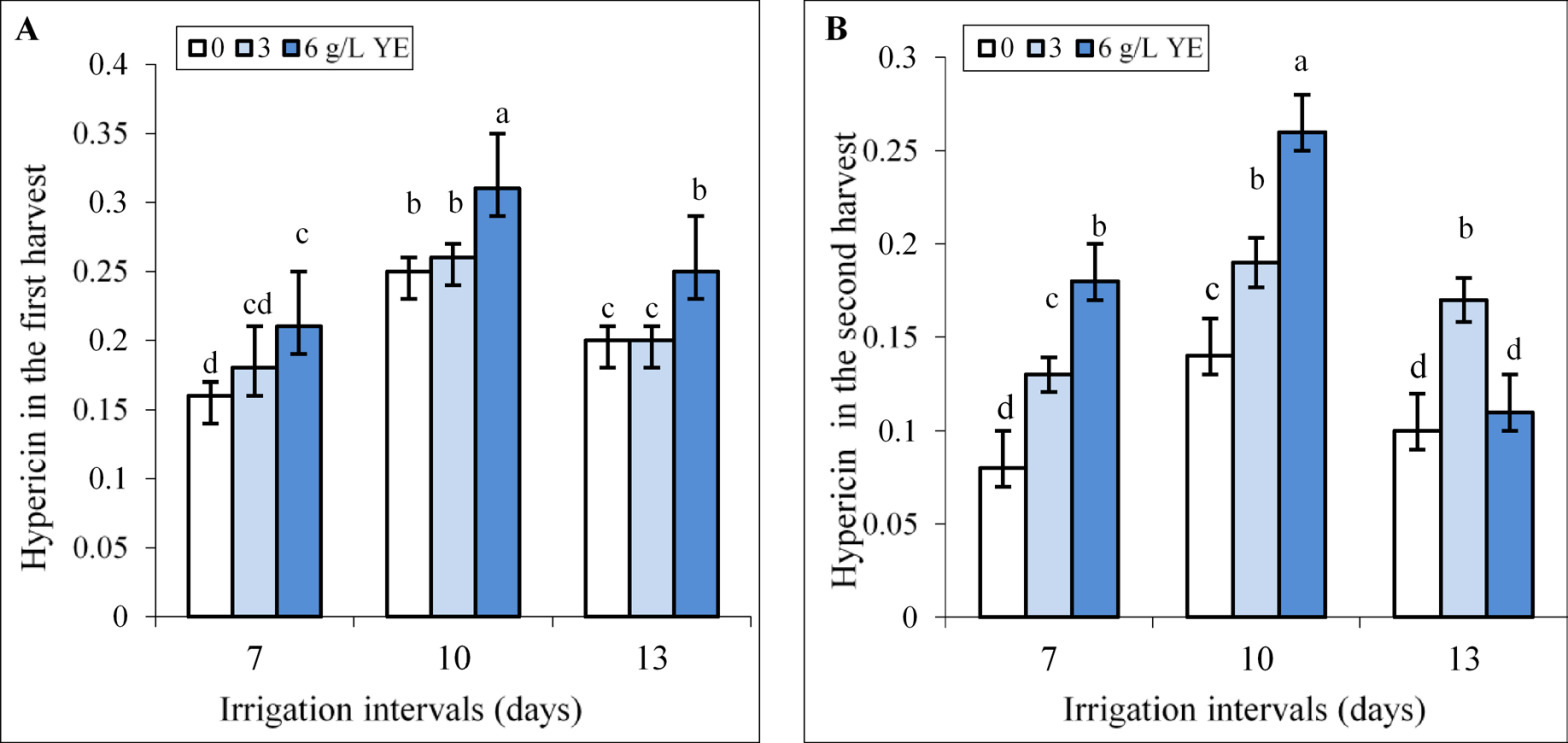
The impact of foliar-applied yeast extract (0, 3, and 6 g L^−1^) on hypericin content in leaves of *Hypericum perforatum* under various irrigation intervals (7, 10, and 13 days). Values with the same letter have no significant difference at *P* ≤ 0.05 based on Duncan’s multiple-range tests.

Our results showed that by increasing the irrigation intervals to 10 days the content of hypericin increased by 56.25% and 75% in the first and second harvests respectively. However, by increasing the irrigation interval to 13 days, hypericin content was less than the 10-day interval in the first harvest and there is no significant difference relative to the respective control in the second harvest (Fig. 6A, B). In the first harvest, the effect of 3 g L^−1^ yeast extract was insignificant, while foliar spraying with the concentrations of 6 g L^−1^ yeast extract increased hypericin content compared to respective controls under all irrigation levels. Foliar spraying with 6 g L^−1^ increased hypericin content by 24.26% and 25% compared to respective controls under mild and severe water deficit. In the second harvest, both concentrations of 3, and 6 g L^−1^ significantly increased hypericin content under all irrigation levels, except in plants treated with 6 g L^−1^ yeast extract and a 13-day irrigation interval which was equal to the respective control (Fig. 6A, B). Foliar spraying with 6 g L^−1^ increased hypericin content by 1.8 fold compared to respective controls under mild water deficit.

### **Interactive effect of yeast extracts and water regimes on TPC and TFC**

**Fig. 7:**
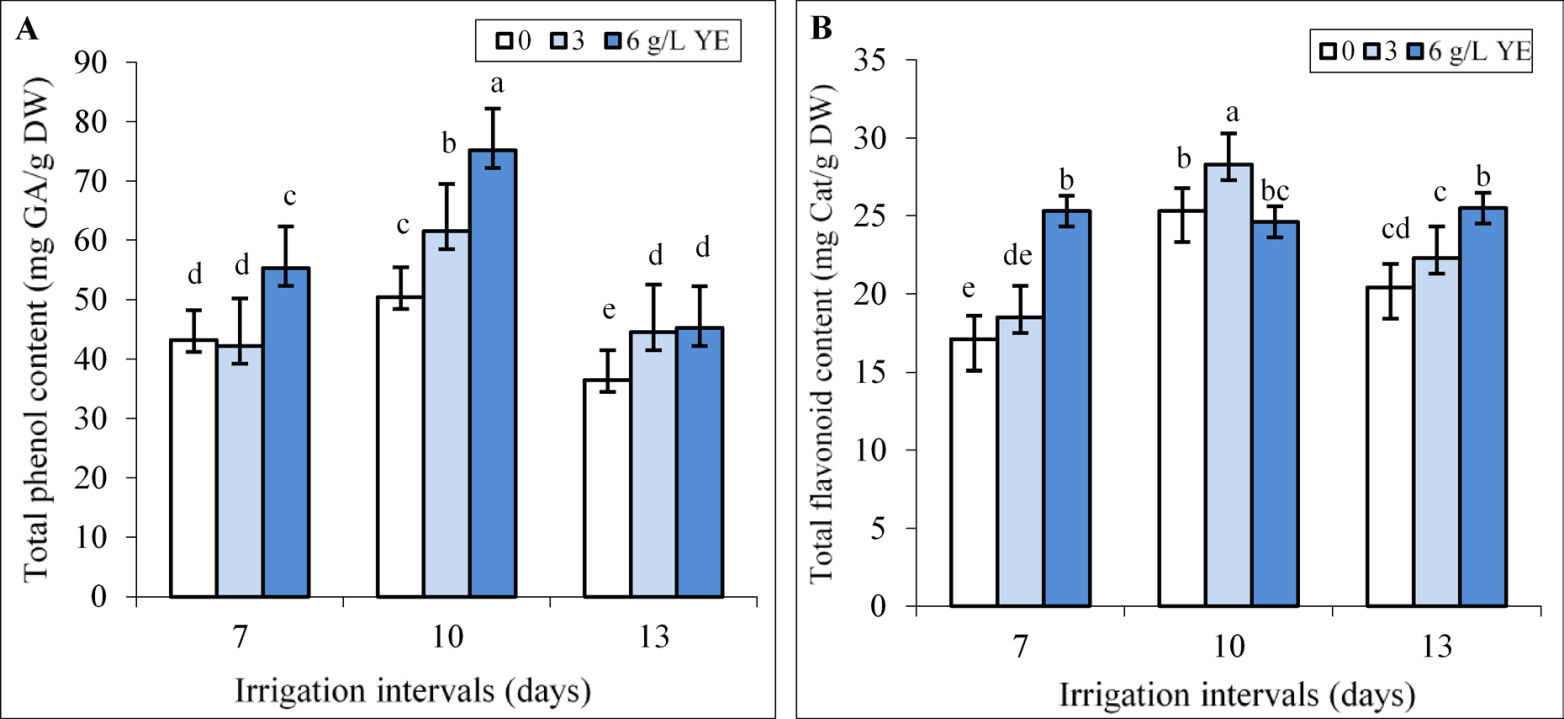
The impact of foliar-applied yeast extract (0, 3, and 6 g L^−1^) on TPC (**A**) and TFC (**B**) in leaves of *Hypericum perforatum* under various irrigation intervals (7, 10, and 13 days). Values with the same letter have no significant difference at *P* ≤ 0.05 based on Duncan’s multiple-range tests.

Under normal irrigation (7-day-interval), spraying with 3 g L^−1^ yeast extract did not change TPC and TFC, whereas significant increments of TPC and TFC were recorded in plants sprayed with 6 g L^−1^ yeast extract (Fig. 7A, B). While increasing the irrigation intervals to 10 days increased TPC and TFC, these attributes in the 13-day interval significantly were less compared to mild water deficit conditions. Both yeast concentrations significantly increased TPC and foliar spray with 6 g L^−1^ yeast extract augmented TPC by 49.2% and 25.8% under mild and severe water deficit (Fig. 7A) respectively. The significant increments of TFC (Fig. 7) were recorded by applying 3 g L^−1^ yeast extract under mild water deficit (11.8%) and foliar spraying with concentration of 6 g L^−1^ yeast under severe water deficit (25%).

### **Interactive effect of yeast extracts and water regimes on DPPH scavenging capacity and FRAP**

**Fig. 8:**
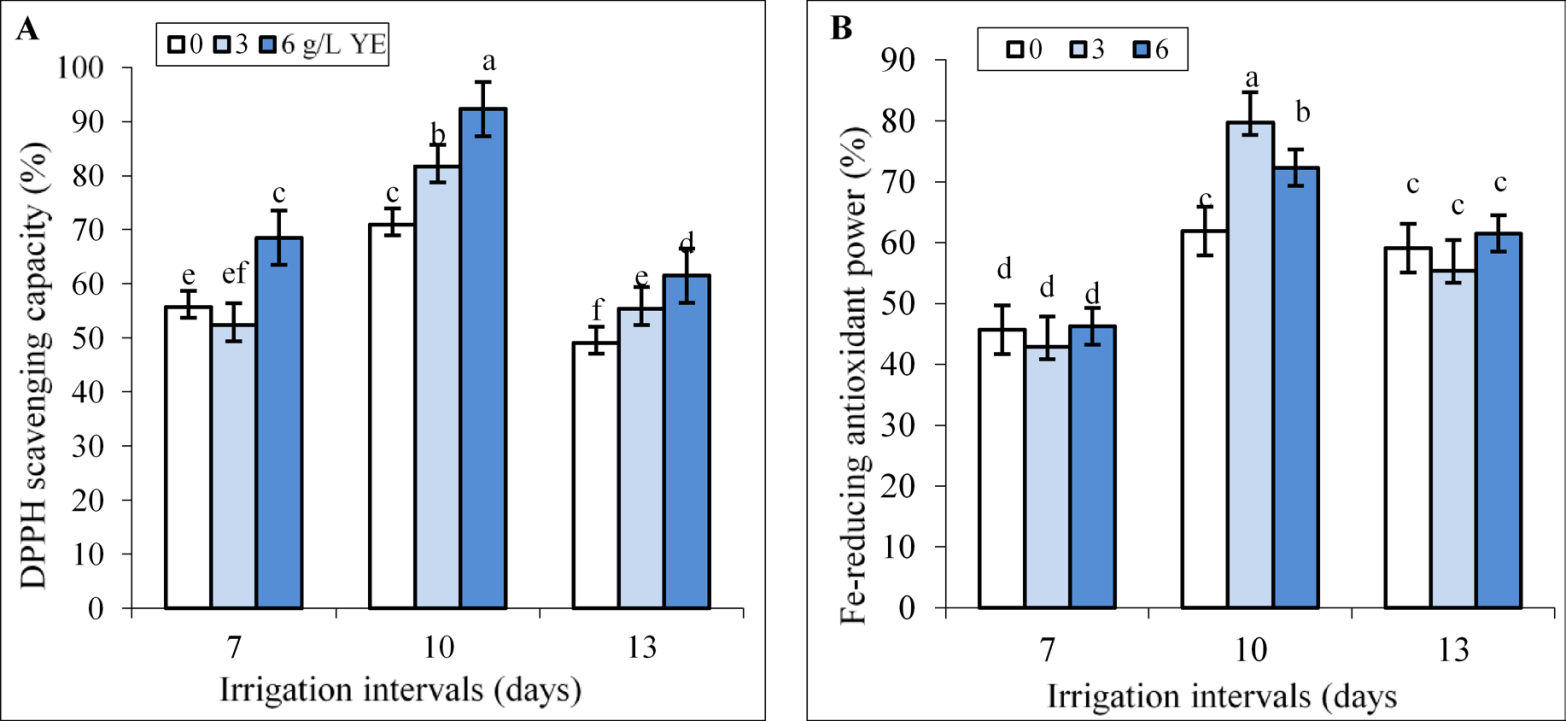
The impact of foliar-applied yeast extract (0, 3, and 6 g L^−1^) on DPPH scavenging capacity (**A**) and FRAP (**B**) in leaves of *Hypericum perforatum* under various irrigation intervals (7, 10, and 13 days). Values with the same letter have no significant difference at *P* ≤ 0.05 based on Duncan’s multiple-range tests.

While spraying with 6 g L^−1^ yeast extract increased DPPH scavenging capacity under normal irrigation (7-day interval), the effect of 3 g L^−1^ yeast extract was insignificant compared to the respective control. Foliar spraying with 3 and 6 g L^−1^ yeast extract did not change FRAP under the 7-day irrigation interval (Fig. 8A, B).

Under mild water deficit, significant increments of DPPH scavenging capacity and FRAP were recorded in plants sprayed with 3 and 6 g L^−1^ yeast extract (Fig. 8A, B). When the irrigation interval increased to 13 days, spraying with 3 and 6 g L^−1^ yeast extract significantly increased DPPH scavenging capacity while having no significant effect on FRAP (Fig. 8A, B). Foliar spraying with 6 g L^−1^ increased DPPH scavenging capacity by 30.18% and 25.25% compared to respective controls under mild and severe water deficit.

### Multivariate analysis

**Fig. 9:**
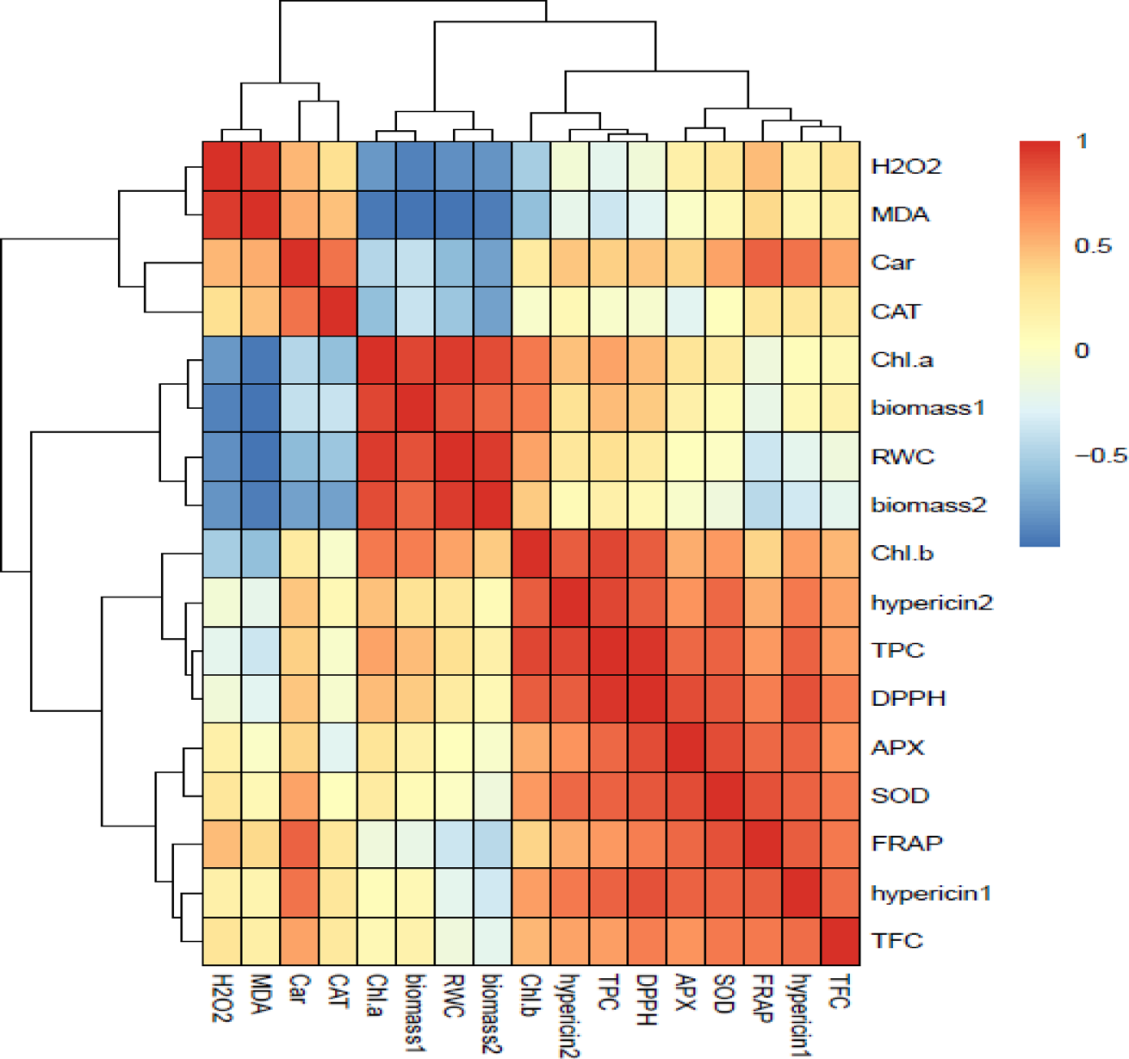
Heat map based on Pearson’s correlation coefficient correlations between all variables in *Hypericum perforatum L.* Strong positive and negative correlations are represented by dark red and dark blue colors, respectively. The investigated variables include: biomass in harvest 1, 2 (biomass1, 2), hypericin in harvest 1, 2 (hypericin 1, 2), chlorophyll *a*,* b* contents (Chl *a*, Chl *b*), malondialdehyde content (MDA), hydrogen peroxide concentration (H_2_O_2_), Ascorbate peroxidase (APX), Catalase activity (CAT), superoxide dismutase activity (SOD), total phenol content (TPC), total flavonoid content (TFC), DPPH scavenging capacity (DPPH) and ferric reducing antioxidant power (FRAP). The correlation heat map based on the Pearson correlation coefficient was performed using R software (version: 3.5.0, http://www.r-project.org).

The heat map obtained based on Pearson’s correlation between traits (Fig. 9) revealed that RWC positively and MDA and H_2_O_2_ contents negatively were correlated with biomass in harvest 1, 2, Chl *a*, and *b* contents. There was a strong and negative correlation between RWC with MDA and H_2_O_2_ contents. In contrast, the MDA and H_2_O_2_ contents positively correlated with TFC, FRAP, hypericin 2, SOD, CAT, APX and carotenoids. Additionally, there was a positive correlation between TPC, TFC, and hypericin 1, 2 with FRAP and DPPH scavenging capacity in plants.

**Fig. 10:**
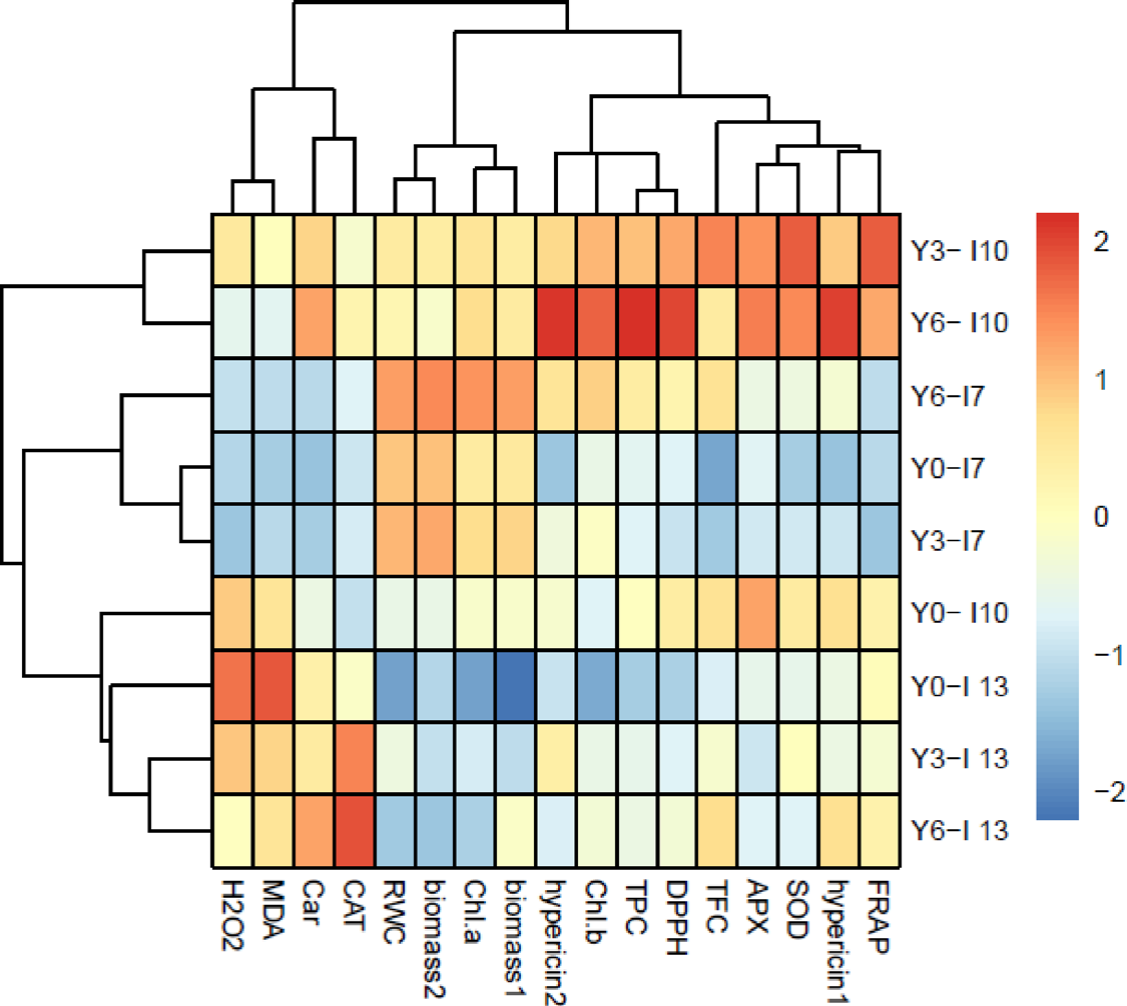
Visualization of the interactions between treatments and variables via a hierarchically clustered heat map. Please see the abbreviation of variables in the capture of Fig. 8. The treatments were included: control (Y0-I7), 3 g L^−1^ yeast extract + 7-day irrigation interval (Y3- I7), 6 g L^−1^ yeast extract + 7-day irrigation interval (Y6- I7), without yeast extract + 10-day irrigation interval (Y0- I10), 3 g L^−1^ yeast extract + 10-day irrigation interval (Y3- I10), 6 g L^−1^ yeast extract + 10-day irrigation interval (Y6- I10), without yeast extract + 13-day irrigation interval (Y0- I13), 3 g L^−1^ yeast extract + 13-day irrigation interval (Y3- I13), 6 g L^−1^ yeast extract + 13-day irrigation interval (Y6- I13). A hierarchical cluster analysis (HCA) between treatments and variables were performed using R software (version: 3.5.0, http://www.r-project.org).

The results of hierarchical clustering analysis (HCA in Fig. 10) showed that the concentration of 3 and especially 6 g L^−1^ yeast extract increased biomass in harvest 1, 2, Chl *a* content, and RWC under normal irrigation (Y3-I7, Y6-I7). While, these yeast extract concentrations further affected the activities of antioxidant enzymes, TPC, TFC, hypericin 1, 2, FRAP, and DPPH scavenging capacity under mild water deficit (Y3-I10, Y6-I10). However, these yeast extract concentrations reduced H_2_O_2_ and MDA contents under severe water deficit by increasing carotenoids and CAT activity (Y3-I13, Y6-I13).

## Discussion

As global water resources diminish, researchers and farmers are seeking innovative strategies to improve plant resilience to water deficit stress while maintaining productivity and even enhancing the quality of medicinal plants^11^. This study for the first time explored yeast extract spraying as a promising approach to improve water deficit tolerance and enhance hypericin and phenolic compounds in St John’s Wort plants.

Water stress reduces soil water potential, complicating water absorption and leading to stomatal closure to minimize transpiration. This process limits CO_2_ influx, suppresses photosynthesis, and ultimately inhibits plant growth and biomass production^47^, as was observed for St John’s Wort in both harvests (Fig. 1). In the second season, plants experienced higher temperatures and lower precipitation (Table 1), thereby water deficit-induced declines in biomass were more pronounced in the second harvest compared to the first. However, foliar application of yeast extract, significantly increased biomass in well-watered and water-stressed St John’s Wort plants in both harvests (Fig. 1A, B). The biostimulant effect of yeast extract is attributed to its phytohormones, such as cytokinins, gibberellins, and auxins, which promote cell division, root and shoot growth, and overall plant development^22,40,48^. Additionally, yeast extract is rich in essential minerals, amino acids, and vitamins that enhance growth and stress tolerance^20,24,37,49^. Previous studies also revealed the positive effect of yeast extract in improving the growth parameters of *Melissa officinalis*L^50^ and milke thistle^37^ under non-stress conditions and in Leucaena plants under salinity stress^48^. Bertea et al.^51^ reported that yeast treatment increased water potential in stems and photosynthetic pigment contents in leaves and enhanced water deficit tolerance in tomato by adjusting ABA level in plant.

The positive correlation between RWC and TChl with growth parameters, (Fig. 9) highlights the critical role of RWC and photosynthetic pigments in sustaining the biomass of St John’s Wort plants. A similar trend was observed in *H. perforatum*^39^, *Satureja rechingeri* Jamzad^52^ and *Foeniculum vulgare* Mill^13^. where water deficit reduced plant growth due to decreased RWC and TChl content. The positive effect of yeast extract on biomass under all irrigation intervals (Fig. 1A) was closely associated with enhancements in RWC (Fig. 2) and an increase in photosynthetic pigment concentrations in leaves (Fig. 3). These findings align with the results reported by Abdelaal et al.^53^ where yeast extract treatment at a concentration of 4 g L^−1^ improved leaf number and area, enhanced RWC, and elevated chlorophyll levels, ultimately leading to increased grain yield and seed oil production in corn under water deficit conditions. Reduced irrigation typically limits water uptake, decreasing cell turgidity and expansion^11,12^. Our previous study showed that yeast extract treatment increased proline content in *H. perforatum* under water deficit^54^. Considering the role of proline in osmotic adjustment^13^ the application of yeast might be positively influenced water balance in plants by increasing the production of osmoprotectants such as proline as reported in garlic plants^40^.

Reduced irrigation significantly decreased chlorophyll *a* and *b* contents in the leaves (Fig. 3A, B), resulting in a diminished supply of photoassimilates necessary for biomass production. Water deficit inhibits the activity of enzymes involved in chlorophyll biosynthesis and promotes ROS-mediated degradation of chlorophyll or the destruction of light-harvesting protein complexes^12^. In St John’s Wort leaves, the concentration of carotenoids increased under a 10-day irrigation interval as a protective mechanism to preserve chlorophyll. However, extending the irrigation interval to 13 days surpassed the plant’s tolerance threshold, leading to carotenoid degradation (Fig. 3C). Foliar application of yeast extract improved these photosynthetic pigments across all irrigation regimes (Fig. 3A, B), indicating its potential to support photosynthetic efficiency even under suboptimal water availability. Consistent with our findings, foliar yeast extract application increased chlorophyll and carotenoid levels and biomass production in *Lippia alba* under non-stress conditions^55^ in cowpea under water deficit^26^ and in lupine plants under salt stress^56^. Yeast extract as a natural source of cytokinins delays leaf senescence and promotes the biosynthesis of α-aminolevulinic acid, a precursor in the chlorophyll biosynthetic pathway^21,26,57–59^. Additionally, yeast extract increased carotenoid content, which can protect chlorophyll by mitigating singlet oxygen^1^O_2_) production and reducing triplet chlorophyll formation, thereby minimizing chlorophyll degradation in stressed leaves^60^.

Water deficit disrupts respiration and photosynthesis, leading to ROS production and increases lipid peroxidation in plants^12^ as verified by the heightened levels of H_2_O_2_ and MDA contents in leaves of St John’s Wort plants (Fig. 4A, B). Under mild water deficit, the activities of APX, CAT, and SOD enzymes (Fig. 5) as well as TPC and TFC (Fig. 7) increased to overcome the ROS-triggered damage in the leaves. However, severe water deficit surpassed the herb’s tolerance levels, resulting in reduced activity of antioxidant enzymes and a decline in TPC and TFC. In other word, the potential of the antioxidant system was not enough to attenuate oxidative stress and consequently H_2_O_2_ and MDA contents increased under severe water deficit (Fig. 4). Foliar application of yeast extract, particularly at the highest concentration (6 g L^−1^), markedly reduced these oxidative stress markers under reduced irrigation. This beneficial effect correlated to enhanced activities of antioxidant enzymes along with an elevated concentration of phenolic compounds in the leaves (Figs. 9 and 10). These findings underscore the potential of yeast extract in strengthening the plant’s antioxidant defense mechanisms to neutralize ROS and reduce lipid peroxidation. Foliar spraying enhanced activities of antioxidant enzymes such as SOD and CAT under mild water deficit conditions (Figs. 5 and 7). This aligns with the findings of prior studies, where foliage-applied yeast extract enhanced antioxidant enzyme activities and reduced lipid peroxidation in drought-stressed crops like maize, wheat^61^ and garlic^40^. However, yeast extract could not increase SOD and APX activity at a 13-day irrigation interval while decreasing ROS and MDA contents in this water regime. This can be explained by the enhanced CAT activity and the heightened phenol and flavonoid contents in these plants. Abdel Latef et al.^62^ also reported that yeast extract alleviated oxidative stress in salt-stressed maize by boosting phenolic compound levels and enhancing the activity of CAT, APX, and SOD enzymes. Likely, hormones or bioactive compounds in yeast extract could activate signaling pathways, leading to increased expression of genes associated with antioxidant responses^63^. Future studies should provide stronger evidence for this hypothesis.

In the present study also the foliage-applied yeast extract, particularly at the highest concentration (6 g L^−1^), increased TPC, TFC, and hypericin content in leaves even under normal irrigation conditions (Figs. 6 and 7). This indirectly suggesting that yeast extract as a bio-elicitor stimulates phenylpropanoid metabolism, which is crucial for the production of phenolic acids and flavonoids. These compounds not only play a role in the biosynthesis of hypericin but also contribute to antioxidant activity, as evidenced by increased DPPH scavenging capacity and FRAP (Fig. 7). It has been demonstrated that yeast extract can activate many genes and enzymes in the phenylpropanoid pathway, such as phenylalanine ammonia-lyase (PAL) and tyrosine ammonia-lyase (TAL)^19,31,32,35^. For instance, yeast extract increased *de novo* biosynthesis of anthocyanin in *Vitis vinifera* L. through upregulating transcription of several genes involved in its biosynthesis pathway^64^. The hypericin biosynthetic pathway depends on photosynthesis and is involved in carbohydrate metabolism and fatty acid metabolism in the upstream steps, while in the downstream steps needs electron-paired donors such as proper flavonoids^65^. Therefore, the increased chlorophyll content (Fig. 3) and photosynthetic efficiency induced by yeast extract might provide greater access to carbon precursors essential for hypericin and phenolic compound production. Our findings agreed with those of Taha et al.^66^ who reported that foliar-applied yeast extract increased chlorophyll and carotenoid contents, growth, total soluble sugars, NPK, resulting in increased phenol and flavonoid contents in leaves of *Azadirachta indica*. However, the effects of yeast extract may be species-specific, as noted by Tóbiás et al.^25^ who observed yeast extract increased chlorophyll content and DPPH scavenging capacity in *Eruca sativa* L. and tomato plants but had no significant impact on lycopene content, TPC, or FRAP in tomatoes.

Phenolic compounds, known for their antioxidant properties, play a crucial role in scavenging ROS under various stress conditions^6,7,9,14,15^. It is also known that, the increased accumulation of hypericins and hyperforin in *Hypericum* plants usually signalizes stressful environmental conditions including the intensity and quality of light, temperature fluctuations and water deficit^5,65^. For example, the production of hypericin increased in *H. perforatum* and *H. adenotrichum* by the addition of polyethylene glycol (PEG) or sucrose to culture media^67,68^. Despite few studies indicating hypericin accumulation is stimulated by biotic and abiotic stresses, there is lack of research on hypericin’s role in plant stress tolerance^5^. In our study, correlations between hypericin content, TFC, and TPC with H_2_O_2_ and MDA levels suggest these compounds are crucial in mitigating oxidative stress induced by water deficit in St John’s Wort plants. Moderate water deficit was observed to increase hypericin content and phenolic concentration (Figs. 6 and 7), thereby enhancing antioxidant defense mechanisms, as evidenced by elevated DPPH scavenging capacity and FRAP values (Fig. 8). Similar findings were reported by de Abreu and Mazzafera^69^ who noted an increase in isouliginosin B, betulinic acid, and flavonoids like rutin and quercetin in *Hypericum brasiliense* under water deficit. According to the growth-defense trade-off theory^70^ moderate stress may reduce plant growth, potentially redirecting carbon allocation toward the production of secondary metabolites as a defensive strategy. However, severe water deficit resulted in decreased hypericin content, TPC and TFC (Figs. 6 and 7), accompanied by heightened oxidative stress (Fig. 4). This is consistent with the findings of Zobayed et al.^71^ who observed that water stress decreased PSII photochemical efficiency and reduced the levels of pseudohypericin, hypericin while increased hyperforin concentrations in leaves of *H. perforatum*. Likely, closing stomata induced by severe water deficit limited CO_2_ assimilation and reduced carbon precursor allocation toward secondary metabolism^11^. Additionally, the high oxidative stress associated with severe water deficit might harm enzymes involved in the biosynthesis of hypericin and phenolics, leading to lower DPPH scavenging capacity and FRAP values in leaves. Similar trends were observed in *Dracocephalum kotschyi* Boiss. where moderate salt stress increased TPC, TFC, and antioxidant activity, whereas high salinity caused declines in these parameters^7^. However, the foliar application of yeast extract significantly enhanced the accumulation of hypericin, TPC, and TFC under both reduced irrigation levels. This result was accompanied by improved antioxidant activity, as evidenced by higher DPPH radical scavenging capacity (Fig. 7), alongside reduced levels of H_2_O_2_ and MDA in the plant leaves (Fig. 4). Similarly, yeast extract application improved oxidative stress tolerance and increased phenolic content in other plant species, such as *Solidago virgaurea* grown in alkaline soils^24^ and wheat subjected to drought stress^26^. Yeast extract stimulates the synthesis of endogenous hormones, which in turn promotes the accumulation of secondary metabolites such as phenolic compounds, and flavonoids, and enhances antioxidant activity^25,33,55^. Polyphenols, not only enhance stress tolerance in plants but also are widely acknowledged for their beneficial impacts on human health. These benefits include improvements in lipid profiles, blood pressure regulation, insulin sensitivity, and the reduction of systemic inflammation^72^. Hypericin has gained a lot of attention in the pharmaceutical industry due to treating mild stress-induced depression and metabolic dysfunction, and in photodynamic diagnosis and therapy of cancer^2,5^. Therefore, the enhancement of phenolic compounds, hypericin content, and antioxidant properties through applying yeast extract not only offers economic advantages but also boosts the marketability of this herb.

## Conclusion

This study highlights the potential of foliar-applied yeast extract as a promising strategy to mitigate water deficit stress and enhance the productivity and quality of *H. perforatum*. By improving photosynthetic efficiency and water content, reducing oxidative damage, and stimulating the activity of antioxidant enzymes, yeast extract not only supports plant growth under water deficit but also enhances the biosynthesis of valuable bioactive compounds like hypericin and phenolic compounds. The plants treated with 6 g L^−1^ yeast extract and mild water deficit exhibited the highest hypericin level, TPC, TFC, DPPH scavenging activity, and FRAP value. Future studies should explore the hormonal and signal alterations and the molecular mechanisms underlying yeast extract’s effects on secondary metabolism and its interactions with other environmental factors. While preliminary studies are encouraging, further research on different medicinal plants across diverse agro-climatic conditions would help validate the efficacy of yeast extract on medicinal plants on a broader scale. As global water resources become increasingly constrained, such eco-friendly approaches will be crucial for ensuring the resilience and productivity of medicinal herbs.

## Data Availability

The data that support the findings of this study are available from the second author upon reasonable request.

## Declarations

### Permission to cultivation in farm of Goldaru company

We acquired permission to conduct the study in the research farm of Goldaru Company at Kelishad, Iran.

### Permission to collect samples

The permission to sowing *Hypericum perforatum* L. was acquired from Agricultural and Natural Resources Ministry of Iran. This plant is one of the medicinal plants that is commonly allowed to be cultivated in Iran and is available and known to most people.Therefore, a voucher specimen of this material has been not deposited in a publicly available herbarium. We thank from Dr. N. Kharazian from Shahrekord University that confirmed the identification of the plants.

### Statement on experimental research and field studies on plants

The plants *Hypericum perforatum* L sampled comply with relevant institutional, national, and international guidelines and domestic legislation of Iran.
